# Corona Virus Disease-19 Vaccine–associated Autoimmune Disorders

**DOI:** 10.2478/rir-2022-0019

**Published:** 2022-10-20

**Authors:** Marriam Hussain Awan, Saba Samreen, Babur Salim, Haris Gul, Shahida Perveen, Amjad Nasim

**Affiliations:** 1 Postgraduate Resident in Rheumatology, Fauji Foundation Hospital, Rawalpindi, Pakistan

**Keywords:** autoimmune phenomenon, COVID vaccines, SARS CoV2 vaccination, vaccine-induced autoimmunity

## Abstract

Coronavirus disease is a highly infectious viral disease caused by severe acute respiratory syndrome virus (SARS nCoV2). It was declared a pandemic within a few months of identification of its index case. The spread of COVID-19 across the globe was rampant, overwhelming healthcare systems and crippling global economies. Since the world was caught off guard by the pandemic, vaccine programs had to be rolled out in emergency to curb its spread. Ten vaccines have been granted Emergency Use Authorization thus far. Much of the side effects we know today are post-marketing adverse effects. Most of them are mild like myalgia and injection-site reactions, but a few of them such as post-vaccination autoimmune diseases have alerted the medical community. These include vaccine-induced thrombotic thrombocytopenia, autoimmune hepatitis, myocarditis, and Graves’ disease. We attempt to summarize the diverse autoimmune phenomena reported after COVID-19 vaccination, with an aim to sensitize the medical community so that they can be better equipped in management when confronted with these diseases. This review by no means refutes the potential benefit of COVID-19 vaccination which has consolidated its place in preventing infections and substantially reducing severity and mortality.

## Introduction

Coronavirus disease is a highly transmissible disease caused by severe acute respiratory syndrome coronavirus 2 (SARS CoV2). The first case of novel coronavirus disease was identified in December 2019 in Wuhan, Hubei Province of China. Little did the world know that this was the beginning of one of the deadliest pandemics seen in recent times. The virus continued to spread unabated worldwide, causing the World Health Organization (WHO) to declare it a Public Health Emergency of International Concern on January 30, 2020, and a pandemic on March 11, 2020. As of May 2022, more than 516 million cases have been reported and 6.25 million confirmed deaths as per the statistics provided by the WHO. Europe was the worst-hit region with the maximum number of reported cases, which stands at a staggering 217 million patients, followed by American continents (154 million).^[1]^

The SARS CoV2 belongs to the family of coronaviruses, which is a positive-stranded ribonucleic acid (RNA) virus having a crown-like appearance under an electron microscope, hence the name. Clinical features of corona virus disease-19 (COVID-19) disease could range from an asymptomatic carrier state (17.9%–33.3%) to mild clinical illness with fever, dry cough, shortness of breath to life-threatening respiratory and multiorgan failure.^[2]^ Cytokine storm, one of the most dreaded complications of COVID-19, occurs in the setting of exaggerated host immune response to viral particles, leading to the generation of large quantities of pro-inflammatory cytokines which orchestrate tissue injury, causing multiorgan failure and increased mortality.^[3]^

While the COVID-19 pandemic continued to spread at an alarming rate, the response from the scientific community was equally vehement for developing the right vaccines in order to curb the spread. More than 200 different candidate vaccines are in different stages of development.^[4]^ Four vaccine designs are used to build various vaccines, which differ according to their mechanisms and effectiveness. These include inactivated vaccines, non-replicating adenoviral vector vaccines, messenger ribonucleic acid (mRNA) vaccines, and protein subunit vaccines. As of May 2022, 10 different vaccines have been granted Emergency Use Authorization by the WHO. These include 2 mRNA vaccines (Pfizer–BioNTech and Moderna), 2 protein subunits vaccines (Novavax and COVOVAX), 3 adenoviral vector vaccines (Johnson & Johnson, Oxford–AstraZeneca, and COVISHIELD), and 3 inactivated vaccines (Sinopharm, Sinovac, and COVAXIN).^[5]^ Two vaccines, namely, Pfizer–BioNTech and Moderna, have been granted full approval by the Food and Drug Administration (FDA) authority for use in people older than 16 years and 18 years, respectively.^[6]^ Until now, 11.69 billion doses of coronavirus vaccine have been delivered worldwide, and 8.74 million doses are now administered every day. Overall, 59.5% of the world population has completed the initial protocol, considered fully vaccinated, whereas 5.9% are partly vaccinated. Low-income countries lag behind, where only 15.9% of the population has received at least 1 dose.^[7]^

With the rampant spread of coronavirus across the globe and Emergency Use Authorization granted to vaccines as a desperate measure to combat the disease effectively, various post-marketing adverse effects have surfaced. These range from mild side effects, such as fatigue, malaise, and soreness at the injection site, to major ones, which have sparked considerable concern. In situ development of a new-onset autoimmune phenomenon and flares of already existing autoimmune diseases have been reported after COVID-19 vaccines.^[8]^ Given the emerging evidence of causal relationship between COVID vaccines and autoimmune diseases, we have attempted to summarize autoimmune manifestations involving different organ systems and proposed mechanisms for their development and their causative vaccines.

## Search Strategy

Online databases including PubMed and Google Scholar were searched for the literature on COVID vaccines and autoimmune diseases developing in temporal association to these vaccines. Keywords used in different combinations included COVID vaccines, SARS CoV2 vaccine, autoimmune diseases, autoimmune hepatitis, myocarditis, autoimmune bullous disease, autoimmune thyroid disease, and vaccine induced thrombotic thrombocytopenia. The literature published till March 2022 has been reviewed.

## Mechanism of Immune Activation by Covid Vaccines

Viruses are notorious for causing immune dysregulation, pre-disposing genetically susceptible individuals to autoimmune disease through a wide variety of mechanisms, and COVID-19 virus remains no exception.^[9]^ Autoimmune manifestations following COVID-19 vaccination are being regularly reported. Multiple underlying mechanisms have been proposed for vaccine-induced autoimmunity, but the main mechanisms that have garnered validation include molecular mimicry; upregulation of immunological pathways, leading to vigorous production of pro-inflammatory cytokines; generation of autoantibodies; and the role of adjuvants in triggering immune response.^[8,10]^

Molecular mimicry is the process where similarity between vaccine components and host proteins initiates immune cross-reactivity, causing the immune cells to attack host cells in susceptible individuals. Only a minority of vaccinated individuals manifest autoimmune disease, suggesting a strong association between genetic predisposition and disease development. Certain autoantibodies, such as platelet factor 4 (PF4 antibody), has been found in patients who developed vaccine-induced thrombotic thrombocytopenia and may represent a significant association.^[11]^

## Autoimmune Manifestations Following Covid-19 Vaccination

### Hepatobiliary Manifestations

New-onset autoimmune hepatitis (AIH) after vaccination is a well-documented phenomenon with vaccines such as influenza^[12]^ and Hepatitis A virus (HAV) vaccine.^[13]^ It has also been reported following both infection with SARS CoV2 ^[14]^ and vaccination against it. It is a form of chronic hepatitis of inflammatory origin, which is triggered by environmental factors in genetically susceptible individuals. It is characterized by derangement of liver functions, development of specific antibodies, elevations of serum immunoglobulin levels, and interface hepatitis on liver histology.^[15]^

Bril *et al*.^[16]^ published the first probable case of autoimmune hepatitis in a 35-year-old postpartum woman who developed jaundice and pruritus 13 d after getting vaccinated with Pfizer–BioNTech vaccine. She had hyperbilirubinemia and transaminitis, positive antinuclear antibodies (ANA) and anti double stranded deoxyribonucleic acid antibodies (anti-dsDNA) antibodies, and normal immunoglobulins. A liver biopsy showed findings consistent with autoimmune hepatitis. Resolution of symptoms and normalization of the liver profile with glucocorticoid therapy further affirmed the proposition.^[17]^ This triggered the publication of many similar cases by researchers all over the world suggesting the role of COVID-19 vaccines as a potential trigger for immune-mediated liver injury. Vuille Lessard *et al*.^[18]^ reported the development of AIH in a 76-year-old woman after getting mRNA-1273 vaccine. Londono *et al*.^[19]^, Tan *et al*.^[20]^, and McShane *et al*.^[21]^ followed suit and observed the development of autoimmune hepatitis in association with mRNA Moderna vaccine. Clayton-Chubb *et al*.^[22]^ and Rela *et al*.^[23]^ reported a case of AIH in association with adenoviral vector vaccines.

Palla *et al*.^[24]^ and Suzuki *et al*.^[25]^ described cases of AIH following administration of BNT162b2 (Pfizer–BioNTech mRNA) vaccine, all of which responded dramatically to steroids.

With the myriad of cases of AIH developing after COVID-19 vaccination, it can be ascertained that it is not a mere casualty but a definitive association, and both mRNA vaccines and adenoviral vector vaccines have been implicated.

### Cardiovascular Manifestations

Myocarditis and myopericarditis have been recognized as a rare complication following SARS CoV2 mRNA vaccination. Bozkurt *et al*.^[26]^ gathered data from various case reports and case series of myocarditis published in peer-reviewed journals. They reported that for ≈ 300 million vaccines administered through June 2021, 1226 probable cases of myocarditis/myopericarditis were reported. The affected patients were primarily young males who developed myocarditis with typical symptoms and ECG findings, confirmed by increased cardiac troponins within 2–3 d after receiving the second dose of mRNA vaccines. A total of 40% had abnormal echocardiography findings, and cardiac magnetic resonance (MR) was abnormal in all patients showing late gadolinium enhancement and myocardial edema, consistent with the diagnosis. Almost all patients recovered with or without therapy.

Since then, multiple case series have been published documenting myocarditis following vaccination with mRNA vaccines for SARS CoV2, as depicted in Table 1.

**Table 1:** Case series reporting myocarditis in association with mRNA COVID vaccines

**Case series**	**Cases**	**Male sex (%)**	**Median age (years)**	**Vaccine type**	**Time between last vaccine and symptom onset**	**% of patients with elevated Troponins**	**% of patients with abnormal CMRI**	**% of patients with symptoms resolved**
Montgomery *et al*.[27]	23	100	25	7 Pfizer, 16 Moderna	1–4	100	100	70
Marshall *et al*.[28]	7	100	17	All Pfizer	2–4	100	100	100
Rosner *et al*.[29]	7	100	24	5 Pfizer, 1 Moderna, 1 J&J	2–7	100	100	100
Larson *et al*.[30]	8	100	29	5 Pfizer, 3 Moderna	1–4	100	100	100

CMRI, cardiac magnetic resonance imaging

### Ocular Manifestations

A number of ocular complications have been reported following vaccination with all types of COVID-19 vaccines that are in use, uveitis being the most frequent. Vaccine-induced uveitis, although rare, is a well-documented phenomenon and has sparked considerable interest. Benage and Fraunfelder^[31]^ studied the incidence of vaccine-related uveitis over a period of 26 years in which 289 cases were reported. Most commonly implicated vaccines in decreasing order of frequency are Hepatitis B virus (HBV), Human papilloma virus (HPV), influenza, bacille Calmette-Guerin (BCG), measles mumps rubella (MMR), and varicella zoster virus (VZV) vaccines.

Cases of acute uveitis have been reported in association with SARS CoV2 vaccines. Rabinovitch *et al*.^[32]^ described 21 patients who developed uveitis following the administration of BNT162b2 SARS CoV2 vaccine. The mean age of the patients were 51.3 years, of whom 8 developed uveitis after the administration of the first dose and 13 after the second dose. The mean time from the day of vaccination to the development of uveitis was reported to be 7.5 d. Moreover, ElSheikh *et al*.^[33]^ reported development of uveitis in association with inactivated COVID-19 vaccine, whereas Alhamazani *et al*.^[34]^ documented uveitis following vaccination with BNT162b2 vaccine.

Other than uveitis, diverse ocular complications have been observed post-COVID-19 vaccination. Some of these complications, as reported by Bolletta *et al*.^[35]^ from the ocular immunology unit, Reggio Emilia, Italy, are summarized in Table 2.

**Table 2:** Autoimmune ocular manifestations observed at the ocular immunology unit, Italy

**Number of patients (eyes)**	**Mean age (years)**	**Mean time from first dose to presentation**	**Vaccine type**	**Ocular complication**	**Number of patient**	**Treatment**	**Outcome**
34 (42)	49.8 years	9.4 d (1–30 d)					
			2 BNT162b2 1 ChAdOx1 nCoV19	Herpetic keratitis	3	Antiviral (acyclovir ointment, oral valaciclovir) topical dexamethasone	Complete resolution
			1 BNT162b2, 1 ChAdOx1 nCoV19	Anterior scleritis	2	Topical dexamethasone	Complete resolution
			2 BNT162b2, 1 ChAdOx1 nCoV19, 1 mRNA1273	NGAU	4	Topical dexamethasone	Complete resolution
			BNT162b2	CMV AU	1	Ganciclovir, topical dexamethasone	Complete resolution
			2 BNT162b2, 1 Ad26.n COV2	Toxoplasmosis retinochoroiditis	3	Sulfadiazine–pyrimethamine, oral steroids	Complete resolution
			BNT162b2	VKH disease	2	MMF, steroids	Complete resolution
			BNT162b2, ChADOx1 nCoV19	Pars planitis	2	Oral steroids	Complete resolution
			mRNA1273, BNT162b2	Retinal vasculitis	2	Oral steroids	Complete resolution
			BNT162b2	Panuveitis	1	Steroids, AZA	Complete resolution
			BNT162b2	MEWDS	3	-	Complete resolution
			ChAdOx1 nCoV19	AMN	1	-	Significant improvement
			mRNA1273	CRVO	1	Intravitreal anti VEGF	Mild improvement
			2 BNT162b2, 2 ChAdOx1 nCoV19	BRVO	4	Intravitreal anti VEGF	Partial-significant improvement
			BNT162b2	NAION	1	-	No improvement
			BNT162b2	Uveitic CNV	1	Intravitreal anti VEGF	Partial to significant improvement
			BNT162b2	Myopic CNV	2	Intravitreal anti VEGF	Partial improvement
			BNT162b2	CSR	1	-	Complete resolution

AMN, Acute macular neuroretinopathy; AZA, azathioprine; BRVO, branch retinal vein occlusion; CMV AU, cytomegalovirus anterior uveitis; CNV, Choroidal neovascularization; CRVO, central retinal vein occlusion; CSR, electrocardiogram; MEWDS, multiple evanescent white dot syndrome; MMF, mycophenolate mofetil; NAION, Non-arteritic anterior ischemic optic neuropathy; NGAU, non-granulomatous anterior uveitis; VEGF, vascular endothelial growth factor; VKH, Vogt-Koyanagi-Harada disease

Table 3 shows ocular adverse events observed in association with inactivated COVID vaccines reported by Chen *et al*.^[36]^ from a tertiary care center in southeast China.

**Table 3:** Ocular complications observed after vaccination with inactivated SARS CoV2 vaccines

**Number of patients (eyes)**	**Vaccine type**	**Ocular complications**	**Mean time from first dose to presentation**	**Treatment**	**Outcome**
7 (10)	Inactivated vaccine (Sinopharm, Sinovac)	3 Vogt–Koyanagi–Harada–like uveitis 1 multifocal choroiditis 1 episcleritis 1 iritis 1 acute idiopathic maculopathy	4.9 d	Steroids	Complete resolution

SARS CoV2, severe acute respiratory syndrome coronavirus 2.

### Neurological Manifestations

Bell's palsy and Guillain–Barre syndrome have emerged as the most common neurological complication occurring in temporal association with COVID-19 vaccination. Wan *et al*.^[37]^ described a case series including 44 patients who developed Bell's palsy within 42 d of getting vaccinated with either BNT162b2 vaccine (16 patients) or inactivated CoronaVac vaccine (28 patients).

Like with other vaccines, Guillain–Barre syndrome has also been described in association with SARS CoV2 vaccines. Several case reports^[38,39]^ and case series have since surfaced (Table 4).

**Table 4:** Case series reporting Guillain–Barre syndrome associated with COVID vaccines

**Case series**	**Number of GBS cases reported**	**Vaccine type**	**Interval from vaccine to symptom onset**
Hanson *et al*.^[40]^	45	Ad.26CoV2 (11) mRNA vaccines (34)	1–84 d
Kim *et al*.^[41]^	13	AstraZeneca (8) Pfizer BNT162b2 (5)	4–30 d

Both new-onset myasthenia gravis^[42,43]^ and exacerbation of existing disease have been reported with COVID vaccines.^[44,45]^ Although rare, acute transverse myelitis has also been reported in association with COVID vaccine. Hirose *et al*.^[46]^ described a case of acute transverse myelitis in a 70-year-old man 7 d after receiving mRNA-1273 SARS CoV2 vaccine. The diagnosis was supported with both radiological and laboratory evidence. The patient recovered after treatment with high-dose steroids.

Shin *et al*.^[47]^ and Zuhron *et al*.^[48]^ described very rare cases of autoimmune encephalitis developing in young female individuals 5 d after receiving their first dose of ChAdOx1 nCoV19 vaccine.

Fernandes *et al*.^[49]^ reported 4 patients who developed neurological sequelae in the form of new-onset seizure and insulin-dependent diabetes mellitus (Pfizer–BioNTech), longitudinally extensive transverse myelitis (Pfizer–BioNTech), Guillian Barre Syndrome (GBS) (ChAdOx1 nCoV19 vaccine), and meningitis retention syndrome (ChAdOx1 nCoV19 vaccine).

Kaulen *et al*.^[50]^ described a case series documenting neurological complications in 21 patients occurring in temporal association of either mRNA or ChAdOx1 nCoV19 vaccination. The complications included central nervous system (CNS) demyelinating diseases (*n =* 8), cerebral venous sinus thrombosis secondary to vaccine-induced thrombotic thrombocytopenia (*n =* 3), inflammatory peripheral neuropathy (*n =* 4), myositis (*n =* 3), and one case each of myasthenia gravis, limbic encephalitis, and giant cell arteritis.

### Hematologic Manifestations

COVID-19 vaccines have the potential to initiate autoimmunity in almost all organ systems of the body, and the blood remains no exception. Rare cases of thrombotic thrombocytopenia have been described in association with the adenoviral vector vaccines with the development antibodies to platelet factor 4 as the underlying pathogenesis.^[11]^ The index case of thrombotic thrombocytopenia was reported in February 2021 when a 49-year-old healthcare worker developed thrombocytopenia leading to portal vein thrombosis and pulmonary embolism after receiving the first dose of ChAdOx1 nCoV-19 vaccine. Despite aggressive treatment, the patient succumbed to complications.^[51]^ More cases started pouring in, and soon, the term vaccine-induced immune thrombotic thrombocytopenia (VITT) was coined. See *et al*.^[52]^ published a case serious reporting 12 cases of VITT leading to cerebral venous sinus thrombosis in association with Ad26.COV2 vaccine, and Schultz *et al*.^[53]^ reported 5 cases of VITT from Norway following vaccination with ChAdOx1 nCov19 vaccine.

Acquired hemophilia with hemorrhagic manifestations has been reported after vaccination with adenoviral vector and BNT162b2 Pfizer–BioNTech vaccine.^[54,55]^ Warm Autoimmune hemolytic anemia (AIHA) after administration of mRNA1273 vaccine^[56]^ and hemophagocytic lymphohistiocytosis in association with inactivated SARS COV2 vaccine has also been reported.^[57]^

### Skin Manifestations

Delayed local skin reactions leading to the so-called “COVID arm” have been observed in association with mRNA COVID-19 vaccines. It is a type of benign dermal hypersensitivity reaction manifested as erythema, edema, and induration at the injection site.^[58]^

Much dreaded autoimmune bullous skin diseases including both bullous pemphigoid and pemphigus (vulgaris and foliaceous) have been reported in association with Oxford–AstraZeneca and Pfizer–BioNTech vaccines, respectively.^[59]^ Pérez-López *et al*.^[60]^ and Russo *et al*.^[61]^ have also reported bullous pemphigoid in association with Pfizer–BioNTech vaccine.

Vasculitic skin rash (AstraZeneca vaccine),^[62]^ atypical maculopapular eruption after Pfizer–BioNTech vaccine^[63]^ and cutaneous thrombosis leading to genital necrosis following the administration of the first dose of Pfizer–BioNTech vaccine^[64]^ has also been described.

### Renal Manifestations

Minimal change disease (MCD) is the commonest immune-mediated renal disease developing after vaccination against SARS CoV2. Lebedev *et al.*^[65]^ reported the index case in a 50-year-old man 10 d after receiving the first dose of BNT162b2 Pfizer–BioNTech vaccine. He presented with anasarca and nephrotic range proteinuria (6.9 g/d). Renal biopsy findings were consistent with those of MCD. He received high-dose steroids and achieved complete remission. More cases of MCD emerged in association with Pfizer–BioNTech vaccine,^[66,67]^ making it the most commonly implicated vaccine. Likewise, other vaccines have also been linked to the development of MCD. Holzworth *et al*.^[68]^ reported the first case of MCD in association with mRNA1273 Moderna vaccine. Likewise, cases of MCD after ChAdOx1 nCoV19 vaccine,^[67,69]^ Ad26.CoV2 vaccine,^[70]^ and the inactivated CoronaVac vaccine^[71]^ have also been reported.

IgA nephropathy is the second commonest autoimmune renal manifestation following vaccination against COVID-19. Six new cases and 6 flares of pre-exiting IgA nephropathy have been reported until August 2021. Eight of the cases developed following mRNA1273 Moderna vaccines, whereas 4 others were associated with Pfizer–BioNTech vaccine.^[72]^ Moreover, renal vasculitis,^[72]^ relapse of membranous nephropathy,^[73]^ relapse of IgG4-related nephritis,^[74]^ and acute rejection after renal transplant^[75]^ have also been reported.

### Endocrine Manifestations

Graves’ disease has been reported in association with SARS CoV2 vaccine. Both molecular mimicry and autoimmune/inflammatory syndrome induced by adjuvants (ASIA) have been proposed as the underlying mechanism. Bostan *et al*.^[76]^ reviewed the available literature till February 2022 and found 20 reported cases of graves’ disease. Of these, 9 patients (45%) had a previous history of autoimmune thyroid disease, whereas 11 patients (55%) developed new-onset graves’ disease. Most common culprit vaccine was found to be mRNA vaccine (15 patients, 75%), whereas 4 cases occurred with vector COVID-19 vaccine and 1 case with inactivated vaccine. The median time from vaccination till symptom onset was 8.5 d.

Other than Graves’ disease, the thyroid gland can be a target of autoimmunity in the form of subacute thyroiditis. Cases have been reported in association with inactivated vaccines, mRNA vaccines, and adenoviral vector vaccines.^[77,78]^

### Miscellaneous

antineutrophil cytoplasmic antibodies (ANCA)-associated vasculitis has been reported in the literature in association with all COVID-19 vaccines, mRNA vaccines being the most common culprit. Twenty-nine cases have been identified thus far till February 2022. Most of the patients presented with renal involvement (93.1%) and responded favorably to immunosuppressive therapy.^[79]^ Other forms of vasculitis, notably giant cell arteritis,^[80]^ leukocytoclastic vasculitis,^[81]^ and flares of quiescent IgA vasculitis^[82]^ and cryoglobulinemic vasculitis,^[83]^ have also surfaced after vaccination.

Reactive arthritis after vaccination is rare and scarcely reported in the literature. An *et al*.^[84]^ reported a single case of reactive arthritis in a 23-year-old woman after inoculation with inactivated CoronaVac vaccine. She was treated successfully with intra-articular steroid injection.

Raviv *et al*.^[85]^ reported the case of a 24-year-old male patient of Ashkenazi Jewish descent who developed a facial rash akin to a malar rash of systemic lupus erythematosus (SLE) 2 d after he received the first dose of mRNA Pfizer–BioNTech vaccine. A subsequent workup showed positive ANA, while all other lupus-specific antibodies were negative. He was diagnosed with SLE as per the 2019 ACR/EULAR criteria and treated with hydroxychloroquine. It has been postulated that the development of SLE in this patient is merely coincidental, but the temporal relationship between vaccination and disease onset may suggest a dysregulated immune response after vaccination.

Table 5 summarizes the most frequently reported multisystem autoimmune manifestations observed thus far and the associated vaccines.

**Table 5:** Most common autoimmune phenomenon observed with COVID vaccines

**Autoimmune manifestation**	**Vaccine type**
AIH	mRNA vaccines, adenoviral vaccines
Myocarditis	mRNA vaccines
Uveitis	mRNA vaccines
Guillain–Barre syndrome	mRNA vaccines, adenoviral vaccines
Vaccine-induced thrombotic thrombocytopenia	Adenoviral vaccine (AstraZeneca)
Bullous skin disease	mRNA vaccines, adenoviral vaccines
MCD	mRNA vaccines, adenoviral vaccines, inactivated vaccines
Graves’ disease	mRNA vaccines, adenoviral vaccines
Autoimmune thyroid disease	

Abbreviations: AIH, autoimmune hepatitis; MCD, minimal change disease.

## Conclusion

Keeping in view the aforementioned, the evidence favoring association between coronavirus vaccination and emergence of new-onset autoimmune phenomenon is sufficiently over-bearing. The mechanisms by which coronavirus vaccination induces autoimmunity in genetically susceptible individuals include molecular mimicry, generation of autoantibodies, and role of vaccine adjuvants. Vaccine-induced autoimmunity is capable of attacking almost any organ system of the body, and this notion is supported by a myriad of cases reported in the literature thus far. No standardized treatment for autoimmune diseases triggered by vaccination exists. They have been managed thus far in a similar fashion as their counterparts not linked to vaccination. We have aimed to sensitize the medical community to vaccine-induced autoimmune diseases in order to open a gateway for discussion, research, and treatment strategies so that they can be better equipped when confronted with a similar problem in clinical practice. At the same time, we would like to highlight the rarity of such occurrences, and we encourage mass vaccination against COVID-19 disease. The benefits of vaccination are undeniable and have been observed in the form of reduction in morbidity and mortality associated with the disease, and it has emerged as a cornerstone in our fight against this deadly pandemic.
